# Codon 141 polymorphisms of the ovine prion protein gene affect the phenotype of classical scrapie transmitted from goats to sheep

**DOI:** 10.1186/s12917-017-1036-1

**Published:** 2017-05-04

**Authors:** Timm Konold, Laura J. Phelan, Ben R. Donnachie, Melanie J. Chaplin, Saira Cawthraw, Lorenzo González

**Affiliations:** 1 Animal Sciences Unit, Animal and Plant Health Agency Weybridge, Addlestone, UK; 2 Pathology Department, Animal and Plant Health Agency Weybridge, Addlestone, UK; 3 Central Sequencing Unit, Animal and Plant Health Agency Weybridge, Addlestone, UK; 4 Pathology Department, Animal and Plant Health Agency Lasswade, Penicuik, UK

**Keywords:** Scrapie, Transmission, Goat, Sheep, Clinical picture, Immunohistochemistry, Western immunoblot, *PRNP* genotype, Codon 141, Prion protein

## Abstract

**Background:**

A study to investigate transmission of classical scrapie via goat milk was carried out in sheep: firstly, lambs were challenged orally with goat scrapie brain homogenate to confirm transmission of scrapie from goats to sheep. In the second study phase, milk from scrapie-infected goats was fed to lambs. Lambs were selected according to their prion protein gene (*PRNP*) genotype, which was either VRQ/VRQ or ARQ/ARQ, with or without additional polymorphisms at codon 141 (FF_141_, LF_141_ or LL_141_) of the ovine *PRNP*. This report describes the clinical, pathological and molecular phenotype of goat scrapie in those sheep that progressed to clinical end-stage.

**Results:**

Ten sheep (six VRQ/VRQ and four ARQ/ARQ, of which three FF_141_ and one LL_141_) challenged with one of two scrapie brain homogenates, and six pairs of sheep (ARQ, of which five LL_141_ and seven LF_141_) fed milk from six different goats, developed clinical disease, which was characterised by a pruritic (all VRQ/VRQ and LL_141_ sheep) or a non-pruritic form (all LF_141_ and FF_141_ sheep). Immunohistochemical (IHC) examination revealed that the pattern of intra- and extracellular accumulation of disease-associated prion protein in the brain was also dependent on *PRNP* polymorphisms at codon 141, which was similar in VRQ and LL_141_ sheep but different from LF_141_ and FF_141_ sheep. The influence of codon 141 was also seen in discriminatory Western blot (WB), with LF_141_ and FF_141_ sheep showing a bovine spongiform encephalopathy-like profile (diminished reactivity with P4 antibody) on brain tissue. However, discriminatory WB in lymphoid tissues, and IHC pattern and profile both in lymphoid and brain tissue was consistent with classical scrapie in all sheep.

**Conclusions:**

This study provided further evidence that the clinical presentation and the pathological and molecular phenotypes of scrapie in sheep are influenced by *PRNP* polymorphisms, particularly at codon 141. Differences in the truncation of disease-associated prion protein between LL_141_ sheep and those carrying the F_141_ allele may be responsible for these observations.

**Electronic supplementary material:**

The online version of this article (doi:10.1186/s12917-017-1036-1) contains supplementary material, which is available to authorized users.

## Background

Classical scrapie is a transmissible spongiform encephalopathy (TSE) of sheep and goats that can be transmitted vertically from dam to offspring and horizontally between animals. Horizontal transmission was demonstrated not only between conspecifics but also from sheep to goats under natural conditions [1, 2], whereas natural transmission from goats to sheep has not been reported. One study described the successful transmission of goat scrapie to sheep by intracerebral inoculation of pooled goat brains [3] but this was prior to the development of more sensitive diagnostic methods and before the effect of prion protein gene (*PRNP*) genotype on disease susceptibility was known.

Three codons are considered important regarding scrapie susceptibility in sheep: codon 136, encoding alanine (A) or valine (V), codon 154, encoding arginine (R) or histidine (H) and codon 171, encoding glutamine (Q) or arginine (R). In Cheviot sheep, the most susceptible genotype is VV at codon 136, RR at codon 154 and QQ at codon 171 (VV_136_RR_154_QQ_171_) whereas AA_136_RR_154_RR_171_ sheep are considered highly resistant [4]. There are other *PRNP* polymorphisms, such as phenylalanine (F) instead of leucine (L) at codon 141, which have been associated with atypical scrapie cases [5], and increased survival times were reported in AA_136_ Cheviot sheep with an F_141_ allele, which had been experimentally inoculated with classical scrapie [6]. *PRNP* polymorphisms also affect classical scrapie susceptibility in goats. For example, lysine (K) instead of glutamine (Q) at codon 222 is associated with increased resistance, and methionine (M) instead of isoleucine (I) at codon 142 is linked to an increased survival time [7].

A more recent study demonstrated transmission of classical scrapie from a goat (II_142_) to two of four VV_136_RR_154_QQ_171_ sheep by oral inoculation with placenta [8] but there was very limited information on the disease phenotype.

The study reported here aimed to provide more detailed information about the clinical, pathological and molecular phenotype of caprine classical scrapie in sheep, in particular with respect to *PRNP* polymorphisms at codon 141 that were detected in these sheep. The disease was produced as part of an experiment to assess the infectivity of goat milk following a pilot study to determine susceptibility of sheep to goat scrapie, the results of which were presented separately [9].

## Methods

### Animal studies

For the pilot study to determine susceptibility of sheep to goat scrapie, brain homogenates were prepared from two naturally infected Anglo-Nubian goats [10] according to established methods [11]: G1460 with II_142_ and G1451 with IM_142_ *PRNP* genotypes. All goats were from a herd that was culled to eradicate scrapie in accordance with Regulation (EC) No 999/2001 and its amendments laying down rules for the prevention, control and eradication of certain transmissible spongiform encephalopathies. More details on these goats are provided elsewhere [12, 13].

Brain recipients were 10 Cheviot lambs with *PRNP* genotypes VV_136_RR_154_QQ_171_ (*n* = 6, subsequently called VV) and AA_136_RR_154_QQ_171_ (*n* = 4, subsequently called AA) born from six ewes from our research flock that was established in England to provide classical scrapie-free sheep for research purposes [14]. *PRNP* genotyping was performed as described previously [15] and included polymorphisms at codon 141, which have shown to influence disease in experimentally infected AA sheep [6] (L_141_ or F_141_ subsequently called LL_141_, LF_141_ or FF_141_). When the study started, recipient sheep were purely selected on the basis of their polymorphisms at codon 136 (VV or AA) to determine the most suitable genotype for a goat scrapie transmission study to sheep. As dam and sire of recipient AA sheep were not both homozygous LL_141_ or FF_141_, recipients were LL_141_, LF_141_ or FF_141_.

The lambs were orally challenged within 24 h of birth with 5 g (as 10% *w*/*v* solution in physiological saline) of one of the two goat brain homogenates. Lambs were raised by their dams until weaning at approximately 10 weeks of age in three pens, two of which contained lambs challenged with either G1460 or G1451 brain and one housed a mixture of lambs challenged with G1460 or G1451 brain (Table 1). Lambs from the different pens were mixed from 35 months of age when the number of animals started to decline.

**Table 1:** Animal details

Animal ID	Sex^a^	*PRNP* codon 136 and 141	Source of infection	Donor	Survival time [d]
1418	M/N	VV LL	Brain	G1451	1167
1419	M/N	VV LL	Brain	G1451	1220
1451	M/N	VV LL	Brain	G1451	1452
1424	M/N	VV LL	Brain	G1460	1154
1425	M/N	VV LL	Brain	G1460	1073
1452	M/N	VV LL	Brain	G1460	1158
1473	M/N	AA LL	Brain	G1460	1060
1319	F	AA LL	Milk (82 l)	G1415	1122
1294	F	AA LL	Milk (76 l)	G1427	1243
1293	M/N	AA LL	Milk (76 l)	G1427	1284
1286	F	AA LL	Milk (87 l)	G1383	1285
1329	M/N	AA LL	Milk (63 l)	G1451	1298
1287	F	AA LF	Milk (87 l)	G1383	1325
1321	F	AA LF	Milk (38 l)	G1460	1333
1324	F	AA LF	Milk (57 l)	G1143	1460
1302	M/N	AA LF	Milk (67 l)	G1451	1466
1323	F	AA LF	Milk (57 l)	G1143	1486
1322	M/N	AA LF	Milk (38 l)	G1460	1493
1320	F	AA LF	Milk (82 l)	G1415	1552
1472	M/N	AA FF	Brain	G1451	1429
1476	F	AA FF	Brain	G1451	1464
1471	M/N	AA FF	Brain	G1460	2030

^a^ *F* female, *M/N* male neutered

For the milk transmission study, recipients were six pairs of AA lambs with either LL_141_ or LF_141_ *PRNP* polymorphisms from the same classical scrapie-free flock because the pilot study implied that AA sheep were more suitable as recipients than VV sheep. As mentioned above, uniform genotypes at codon 141 could not be pre-selected in the recipient sheep although this time dams and sire were chosen to produce either LL_141_ or LF_141_ lambs because infection in the pilot study was first demonstrated in AA sheep with an L_141_ allele [9]. The lambs were fed milk from six different scrapie-infected goats (volume 38–87 l per lamb), which were all from the same herd and included the two goats that also provided brains for the pilot study. Once all the goat milk was consumed, lambs were fed milk replacer (Lamlac, Volac International Ltd., Orwell, UK). Pairs of milk recipients were kept separate until scrapie infection was confirmed by rectal biopsy (see below), at which time they were mixed. All sheep were fed straw and concentrates after weaning at approximately 10 weeks of age.

A graphic representation of the study design is provided as Additional file 1: study design.

### Monitoring scrapie infection status

Scrapie infection status was determined by the immunohistochemical examination of biopsies of recto-anal mucosa-associated lymphoid tissue (RAMALT) [16] from 6 months (pilot study) and 9 months (milk transmission study) of age; rectal biopsies were repeated only in sheep with previously negative results.

Sheep were monitored twice daily by farm staff and were systematically examined for neurological signs first at 20 months of age, then at 31 months and then usually monthly or more frequently depending on clinical status, using a short examination protocol for detection of scrapie [17]. A full neurological examination [11] was carried out prior to culling, and an electrocardiogram was recorded as described previously [18] in those sheep where cardiac arrhythmia was suspected during the physical examination. To document progressive pruritic behaviour, a scratch test score was calculated retrospectively. This was the number of consecutive positive responses (unequivocal stereotypical behaviour [19] until culling divided by the total number of clinical assessments ranging from 0 (no response prior to cull) to 1 (response at each assessment until cull). Inconclusive responses [19] counted towards positive reactions if they occurred prior to a positive response because it was interpreted as early sign of pruritus consistent with the progressive nature of scrapie. Thus, an inconclusive response followed by no scratch response on the subsequent examination or a positive scratch test that disappeared prior to culling did not count as consecutive positive response until culling because clinical signs were less likely to be associated with a progressive disease if they disappeared.

Assessments were made blind without knowledge of the genotype or inoculum although the scrapie status was known once animals were mixed. Sheep were euthanased with intravenous injection of secobarbital and cinchocaine (Somulose, Dechra, Shrewsbury, UK) at clinical end-point, which was reached when animals displayed progressive abnormalities in sensation (positive scratch test with or without alopecia, absent menace response) and movement (ataxia, limb weakness, tremor).

### Post-mortem investigation

The brain and a range of lymphoid tissues were taken and processed for immunohistochemistry (IHC) and Western immunoblot (WB) examination.

For IHC, tissue from the central nervous system (CNS) and lymphoid tissue were formalin-fixed, wax-embedded and examined with six different antibodies, which bind to different amino acid (aa) residues of ovine PrP: BG4 (aa 46–54; the Roslin Institute, Edinburgh, UK), 12B2 (aa 93–99, same as P4; Wageningen University & Research, Lelystad, Netherlands), 521.7 (aa 100–102; courtesy of Jan Langeveld, Wageningen Bioveterinary Research, Lelystad, Netherlands), 9A2 (aa 102–104; Wageningen University & Research), BH1 (aa 143–154; the Roslin Institute, Edinburgh, UK) and R145 (aa 222–226, APHA Weybridge, Addlestone, UK) and are used to distinguish classical scrapie from bovine spongiform encephalopathy (BSE) and CH1641-like strains [20, 21]. The discriminatory IHC was applied to nine AA sheep, three each with the different polymorphisms at codon 141, which had similar scrapie-associated prion protein (PrP^sc^) profiles and total amount of PrP^sc^ (see below). A naturally infected classical scrapie sheep, a sheep orally challenged with BSE brain homogenate and a sheep intracerebrally inoculated with CH1641 brain homogenate were used as controls; all three controls were AALL_141_. The robustness of the discriminatory IHC has been previously demonstrated [20].

Lesion profiling was carried out as described previously [6]: tissue sections of the brain at seven levels were immunolabelled with antibody R145 for simplified scoring of PrP^sc^ on a scale from 0 (none) to 3 (striking) in steps of 0.5 and including 0.2 for trace immunolabelling. Five different PrP^sc^ patterns were distinguished: i) intraneuronal, ii) intraglial (intramicroglial and intrastrocytic types combined), iii) extracellular glia-associated (subpial, subependymal, perivascular, stellate and perivacuolar types combined), iv) grey matter neuropil-associated (diffuse particulate, coalescing, perineuronal and linear types combined), and v) other types (ependymal, vascular plaque and non-vascular plaque types combined). The average value for each PrP^sc^ pattern from the seven areas examined was used to create an individual PrP^sc^ profile and, for grouping by genotype, the profiles constituted the mean of the individual sheep profiles within each genotype group.

WB examination for detection of the proteinase-resistant form of PrP^sc^ (PrP^res^) was carried out on samples of the caudal medulla and medial retropharyngeal lymph node using antibodies Sha31 (aa 156–163) and P4 according to the BioRad TeSeE universal WB protocol (BioRad Laboratories, Hemel Hempstead, UK). Parallel testing with the two specific monoclonal antibodies aids in the discrimination between classical BSE and scrapie where Sha31 detects PrP^res^ in both cattle and sheep, whereas P4 is more selective for scrapie PrP^res^ [22]. Additional antibodies used were 12B2, SAF32 (aa 86–91; SPI-Bio, Bertin Pharma, Montigny le Bretonneux, France) and 9A2. Sigma biotinylated molecular mass markers as well as a known UK classical scrapie (ovine VRQ/VRQ and caprine II_142_) brain sample, known UK bovine BSE brain sample and known ovine VRQ/VRQ non-neural mesenteric lymph node sample (for WB on lymphoid tissue) were included as positive controls for profile comparisons. All control samples were homogenates from a single naturally infected animal. All samples were tested once.

From each sample, 0.35 g tissue was ribolysed, purified, treated with proteinase K (following treatment with DNase for all medial retropharyngeal lymph node samples) and PrP^res^ concentrated according to the kit instructions and with the reagents supplied. As four samples produced weak profiles upon initial examination of caudal medulla tissue, these samples were processed in duplicate and the pellets combined (double pellets) for subsequent runs. Samples were heated for 4 min at 100 °C prior to loading on gels. A sample of 15 μl each was loaded in duplicate lanes onto pre-cast 12% bis-tris gels (Criterion, Bio-Rad) and electrophoresed for 50 min at 200 V. The proteins were then transferred onto PVDF membranes (115 V for 60 min) and blocked (Bio-Rad blocking solution for Sha31 and milk powder solution for other antibodies) for 30 min at room temperature. They were probed with the kit primary antibody (Sha31) for 30 min at room temperature and additional antibodies (P4, 12B2, SAF32, 9A2) for 1 h at room temperature.

The membranes were washed, incubated for 20 min in Bio-Rad secondary antibody (Sha31) or goat anti-mouse conjugate (P4, 12B2, SAF32, 9A2) at room temperature, washed again and the membranes were incubated with ECL substrate (Amersham) for 1 min. The signal was detected with the Fluor-S MultiImager (Bio-Rad).

The total amount of PrP^res^ signal emitted from the diglycosylated, monoglycosylated and unglycosylated protein bands was calculated by means of the volume analysis tool in the Quantity One software (Bio-Rad) for antibodies Sha31 and P4. The global background was subtracted and the ratio for the ovine scrapie control sample set at 1; the ratio for the test samples was then calculated relative to the ovine scrapie control sample. The cut off was set at 2.0 and any sample with a ratio below this was considered to be classical scrapie-like and any ratio above this was considered classical BSE-like.

## Results

Animal data, such as *PRNP* genotype, sex and survival time, of the 22 sheep that developed a TSE (ten challenged with brain, 12 challenged with milk) are provided in Table 1. The influence of *PRNP* genotype on survival time has already been discussed in the previous publication [9] and will not be mentioned in this manuscript.

### Clinical signs

**Fig. 1:**
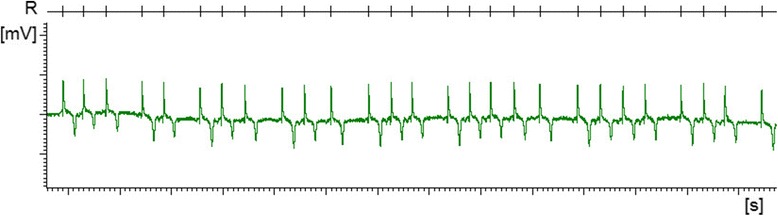
Electrocardiogram recorded from LL_141_ milk recipient 1329 with cardiac arrhythmia. Extract of an electrocardiogram recorded over 5 min prior to cull whilst the animal was restrained in its pen with other sheep; overall heart rate 112 beats per minute. R = R peak intervals; major unit on x axis is second, major unit on y axis is 0.1 mV. Note the irregular rhythm of the R peak intervals, which do not appear to follow a particular pattern

Signs of pruritus, characterised by a stereotypical response to scratching of the back (positive scratch test) and/ or alopecia with or without skin lesions suggestive of excessive rubbing, scratching or nibbling were displayed by all 12 sheep that were either VV or LL_141_ (see Additional file 2 for an example of the pruritic form). Of the four sheep with alopecia (one VV and three LL_141_ sheep), one LL_141_ sheep did not display a clear stereotypical response when scratched although the scratch response had been positive at the examination three days earlier. Scratch test scores ranged from 0.21 to 1.0 (median: 0.39) in VV sheep and 0 to 0.79 (median 0.5) in LL_141_ sheep. None of the LF_141_ or FF_141_ sheep displayed signs of pruritus and the scratch test score was 0 for all sheep with these polymorphisms. By contrast, the most frequent sign observed in LF_141_ or FF_141_ sheep was limb weakness (all three FF_141_ and six of seven LF_141_) in combination with ataxia (two of three FF_141_ and all LF_141_) whereas limb weakness was observed in only one LL_141_ sheep even though most of them (all LL_141_ and four of six VV sheep) were ataxic (see Additional file 3 for an example of the non-pruritic form). A head tremor was present in all six VV sheep but rare in AA sheep; other movement disorders were lip twitches (one FF_141_ sheep, see Additional file 3 showing this sheep with twitching lips) and spontaneous myoclonus (one LL_141_ sheep). Bruxism was audible in five of the six LL_141_ sheep and three of the six VV sheep but only in one FF_141_ sheep. Infrequent signs were cardiac arrhythmia (two LL_141_, three LF_141_, one FF_141_ sheep; see Fig. 1 for an example), lack of menace response (one VV, LF_141_ and FF_141_ sheep), cataplexy-like collapse (one FF_141_, one LL_141_ sheep), inappetence (one LL_141_ sheep)_,_ disequilibrium when blindfolded (two VV sheep and one LL_141_ sheep) and othaematoma (one VV sheep). Table 2 lists the percentage of animals per genotype group that displayed selected clinical signs. Full details of the clinical signs are given in Additional file 4: clinical and pathological data.

**Table 2:** Percentage of sheep with selected clinical signs grouped by genotype

*PRNP* genotype codon 136 and 141	*N*	Positive scratch test	Alopecia	Bruxism	Tremor	Ataxia	Limb weakness	Absent menace response
VV LL	6	100%	17%	50%	100%	67%	0%	17%
AA LL	6	83%	50%	83%	33%	100%	17%	0%
AA LF	7	0%	0%	0%	29%	100%	86%	14%
AA FF	3	0%	0%	33%	33%	67%	100%	33%


Additional file 2:pruritic form of scrapie. The clip shows the clinical signs of ALRQ/ALRQ wether 1293, which was fed milk from scrapie-affected goat G1427 from 20 h after being born. This sheep, first shown at 1222 days of age and indicated briefly by a red circle to distinguish it from its pen mate, falls when turning. There is also excessive sinking in the hind limbs when it turns on the spot. Scratching of the back results in rising of the head and later lip licking (positive scratch test). Sixty days later, at 1282 days of age, there is evident hind limb ataxia, characterised by delayed hind foot placement and crossing of the hind limbs particularly visible when the animal turns. A positive scratch test can be elicited more easily by simply putting pressure on the spine. (MP4 14151 kb)



Additional file 3:non-pruritic form of scrapie. This clip presents the clinical signs of AFRQ/AFRQ ewe 1476 at 1464 days of age following oral challenge on its day of birth with brain from scrapie-affected goat G1451. Twitching of the upper lip (repetitive myoclonus of the lip muscle) is present. There is no evidence of pruritus because this is a fully fleeced sheep without obvious wool loss and it does not respond to scratching of the back (negative scratch test). There is lack of foot placement correction when the sheep is pushed sideways suggestive of proprioceptive deficits and it briefly sits down. Hind limb ataxia is evident with excessive sinking in the hind limbs. (MP4 13735 kb)


### Immunohistochemistry

**Fig. 2:**
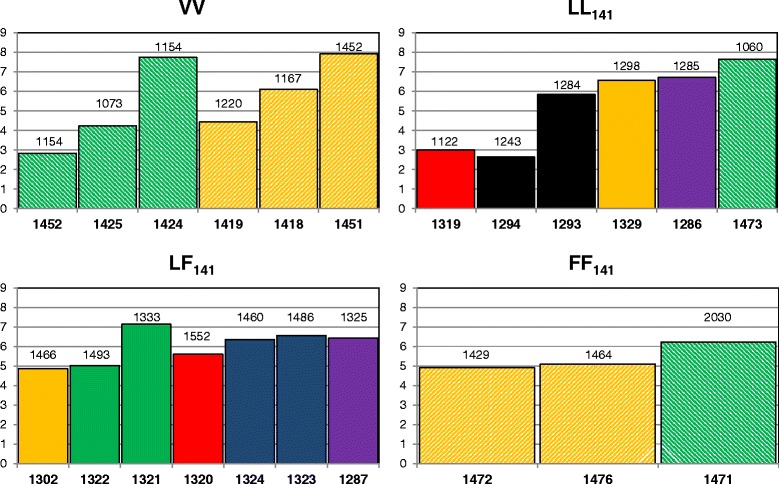
Total magnitude of brain PrP^sc^ in sheep of different genotypes. Solid bars = milk recipients; patterned bars = brain recipients. Colours identify goat donors (same colour = same donor). Survival times are indicated on the top of each bar

Fig. 2 shows the total magnitude of PrP^sc^ immunolabelling in the brain of each sheep, which reflects the sum of all average scores for the different PrP^sc^ patterns. Whilst there was little variation between sheep with an LF_141_ (mean: 6.20, standard deviation [SD]: 0.69) or FF_141_ (mean: 5.41, SD: 0.71) *PRNP* genotype, more variation was seen in VV (mean: 5.55, SD: 2.05) and LL_141_ sheep (mean: 5.40, SD: 2.08) where the total brain PrP^sc^ score in one sheep could be more than double the score in another sheep irrespective of whether they were challenged with brain or milk from the same donors (see 1452 compared with 1424 and 1294 compared with 1293 in Fig. 2).

**Fig. 3:**
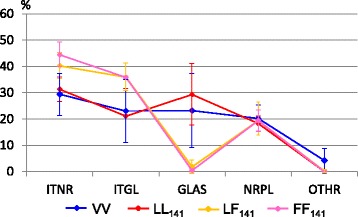
Mean PrP^sc^ profiles in the brains of goat brain and milk recipients sheep of different genotypes. ITNR = intraneuronal, ITGL = intraglial, GLAS = glia-associated, NRPL = neuropil-associated, OTHR = other types of PrP^sc^ immunolabelling. Shown are the mean values with standard error of mean bars

**Fig. 4:**
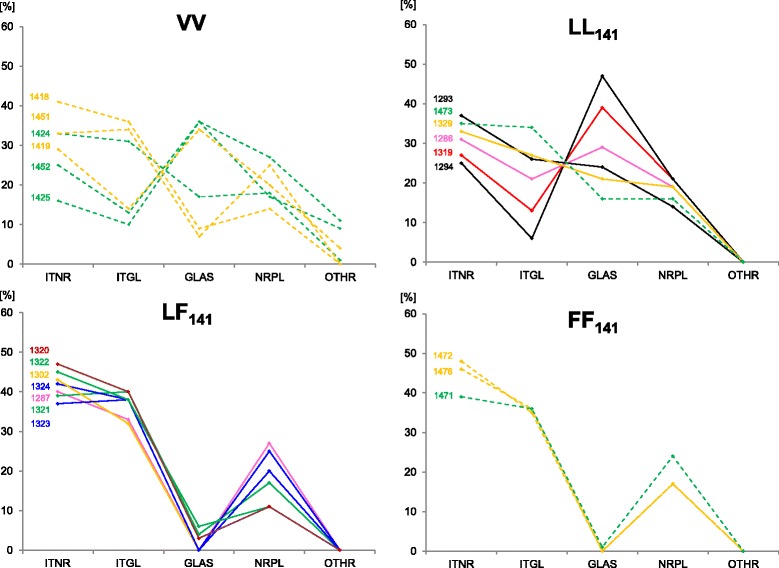
Brain PrP^sc^ profiles of individual sheep. Solid lines = milk recipients; dashed lines: brain recipients. Colours identify goat donors (same colour = same donor). ITNR = intraneuronal, ITGL = intraglial, GLAS = glia-associated, NRPL = neuropil-associated, OTHR = other types of PrP^sc^ immunolabelling

The mean PrP^sc^ profiles of goat brain and milk recipients, separated by *PRNP* genotype, are shown in Fig. 3. The profiles were similar in VV and LL_141_ sheep but differed from the profiles seen in LF_141_ and FF_141_ sheep. When the PrP^sc^ profiles from individual sheep were compared within the *PRNP* genotype group (Fig. 4), differences were apparent between LL_141_ sheep fed milk from different donors but not between LF_141_ sheep, which all displayed similar profiles. Also, challenge with brain from the same donor resulted in profile differences in VV sheep but not in FF_141_ sheep.

**Fig. 5:**
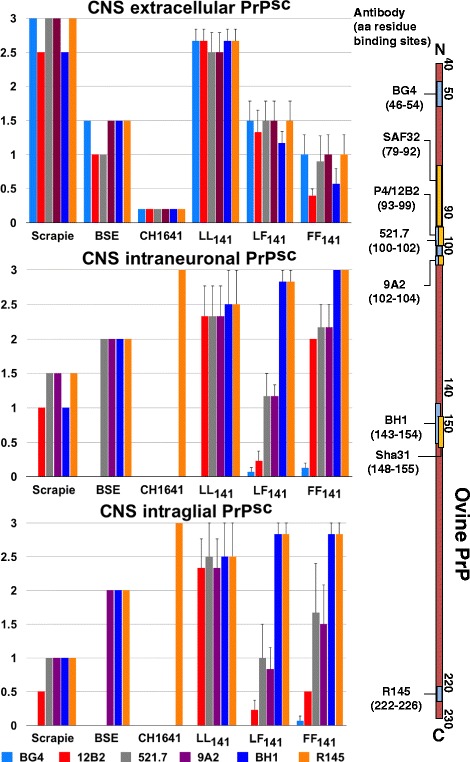
Differences in PrP^sc^ localisation depending on codon 141 polymorphisms observed by discriminatory immunohistochemistry on brain tissue using six different antibodies. Classical scrapie, classical ovine BSE and the CH1641 strain (single animals) are shown for comparison. Shown are the mean values with standard error of mean bars. The diagram on the right represents the ovine prion protein and the binding sites of the antibodies used in this study

Discriminatory IHC using six different antibodies revealed that antibody detection in the different compartments in CNS tissue of AA sheep was dependent on polymorphisms at codon 141 of the ovine *PRNP* (see Fig. 5, which shows the immunolabelling pattern in different compartments using six antibodies with different affinity to ovine PrP^sc^). In lymphoid tissue, all antibodies detected the same levels of PrP^sc^ in macrophages, which was consistent with classical scrapie.

Individual scores are shown in Additional file 4: clinical and pathological data.

### Molecular profiles

**Fig. 6:**
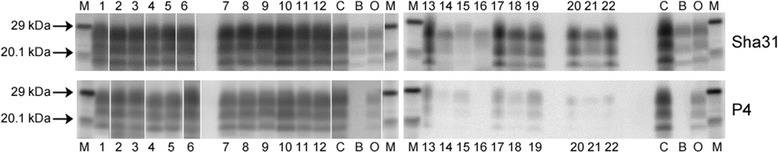
Western immunoblot profile of brains from sheep challenged with caprine scrapie. Lanes 1–6: VV sheep (1: 1418; 2: 1419; 3: 1451; 4: 1452; 5: 1425, 6: 1424); lanes 7–12: LL_141_ sheep (7: 1319; 8: 1294; 9: 1293; 10: 1286; 11: 1329; 12: 1473); lanes 13–19: LF_141_ sheep (13: 1320; 14: 1287; 15: 1321; 16: 1302; 17: 1324; 18: 1323; 19: 1322); lanes 20–22: FF_141_ sheep (20: 1471; 21: 1472; 22: 1476). M: molecular mass markers; C: caprine classical scrapie II_142_; B: classical BSE; O: ovine classical scrapie (VV). For better visualisation, lanes 2, 3, 6 and controls were cropped from the original blots, indicated by the white boundary lines, to create a composite image. Reactivity with the N-terminal antibody P4 was reduced in LF_141_ and FF_141_ sheep, which is unlike classical scrapie

**Fig. 7:**
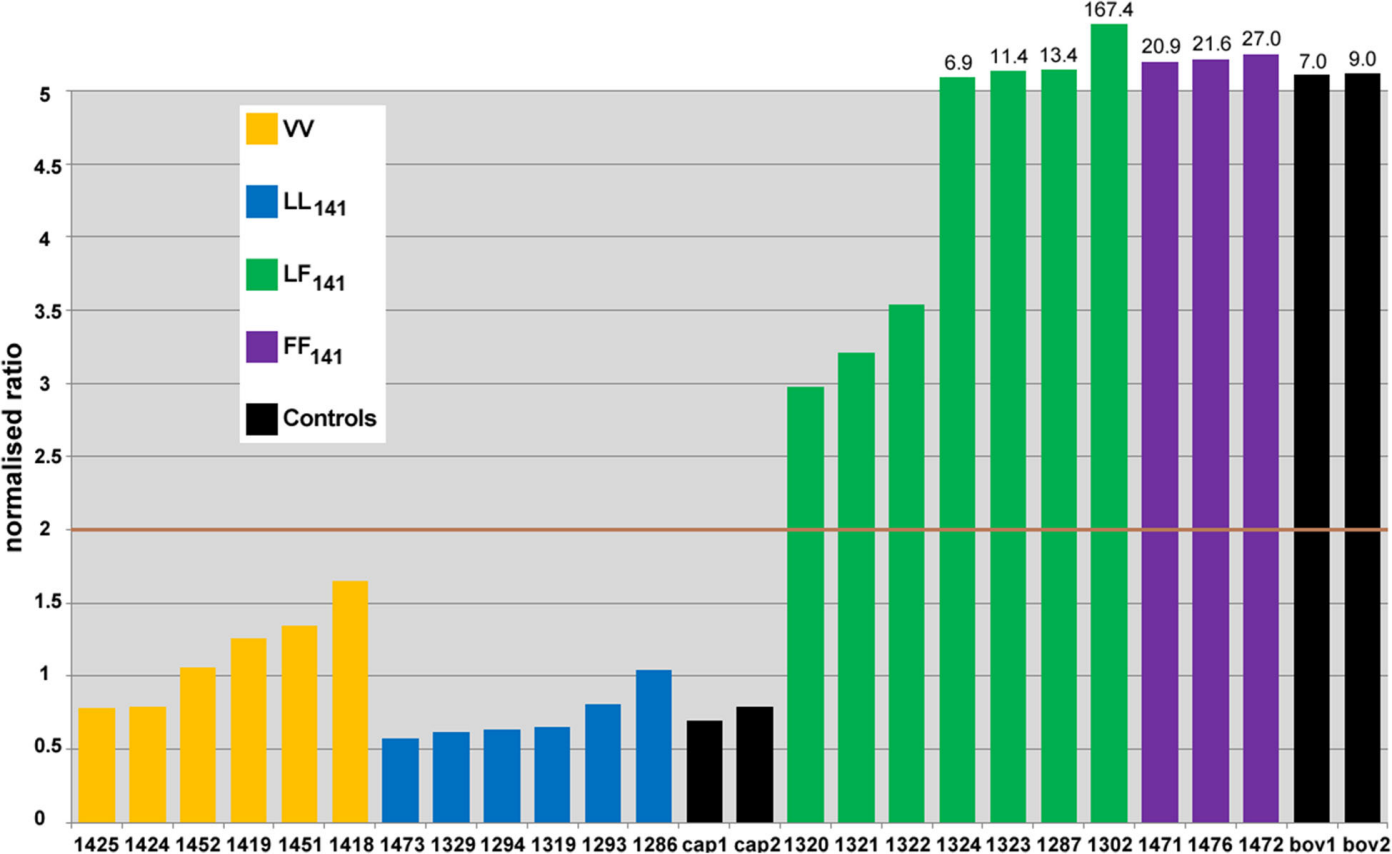
Comparison of the reactivity of Sha31 and P4 antibodies in the Western immunoblot from sheep challenged with caprine scrapie and controls. Animal numbers are displayed under each bar; cap1-2 and bov 1–2 are naturally infected caprine classical scrapie (II_142_) and bovine classical BSE control samples respectively run in duplicates on different gels. The cut-off value of 2 for separation between classical scrapie and BSE is indicated by the brown line. Values above 5 are not to scale and are displayed above the respective bar. LF_141_ and FF_141_ sheep had antibody ratios above 2, similar to the bovine classical BSE control samples

Molecular profiles obtained by WB testing of caudal medulla with antibodies Sha31 and P4 are shown in Fig. 6. Differences were seen in relation to the reactivity with the N-terminal antibody P4, which was dependent on *PRNP* polymorphisms at codon 141: PrP^res^ was detected with this antibody in VV and LL_141_ sheep, similar to the classical scrapie ovine and caprine control samples, whereas reactivity was reduced in LF_141_ sheep and greatly reduced or absent in FF_141_ sheep, similar to the classical BSE control sample. Similar observations were made with two further N-terminal antibodies, 12B2 and SAF32. The Sha31/P4 antibody ratios were less than 2 for VV and LL_141_ sheep as seen with the classical scrapie control samples, but above 2 in LF_141_ and FF_141_ sheep. In fact, the ratio was above 5 in 4 of 7 LF_141_ sheep and in all FF_141_ sheep (Fig. 7). By contrast, WB reactivities to Sha31 and P4 antibodies on medial retropharyngeal lymph node samples were similar for all sheep, regardless of the 141 polymorphism, and similar to the classical scrapie control samples.

## Discussion

Although this study was originally carried out to investigate the transmission of scrapie via goat milk using sheep as recipients it has provided valuable information about the influence of the *PRNP* genotype, particularly at codon 141, on the clinical, pathological and molecular phenotype of goat scrapie in sheep. It has been demonstrated previously that polymorphisms at codon 141 may affect survival time and disease phenotype in sheep on cross-*PRNP* genotype inoculations with pooled sheep brain homogenate [6].

Clinically affected sheep presented with a range of clinical signs, which have been reported previously in sheep with scrapie and indicated that caprine scrapie transmitted to sheep produced a clinical disease associated with scrapie in sheep. This included less common signs such as cardiac arrhythmia, cataplexy-like collapse and othaematoma, possibly as a result of excessive pruritus, which have nevertheless been reported in natural scrapie in sheep [18, 23–26]. Other involuntary movements, such as myoclonus (brief shock-like jerks due to sudden involuntary contraction of one or more muscles [27]) have been reported in some cases of scrapie [28, 29] but are usually more frequent in BSE in cattle or Creutzfeldt-Jakob disease in humans and often in response to an external stimulus (startle myoclonus) [30–32].

Clinical disease in sheep may vary according to breed, region and country, presenting either as pruritic or non-pruritic form [33]. The results presented here clearly demonstrate that the clinical phenotype is affected by *PRNP* genotype, notably polymorphisms at codon 141, of sheep of the same breed. All LL_141_ sheep (regardless of donor goat and *PRNP* genotype at codon 136, VV or AA) exhibited the pruritic form, which is also the predominant form in sheep infected with the BSE agent and characterised by a positive scratch test (lip licking or head movements when the back is scratched), excessive rubbing and scratching resulting in wool loss [19]. By contrast, LF_141_ and FF_141_ sheep presented with the nervous form, characterised by ataxia and limb weakness without any signs of pruritus, regardless of donor goat, which is clinically unlike experimental BSE in sheep. These results provided further evidence that the clinical presentation is dependent on the *PRNP* genotype as suggested by studies in naturally infected sheep where strain as confounding factor could not be excluded [29, 34].

The results also provided further evidence that the pathological phenotype was influenced by the *PRNP* genotype at codon 141. PrP^sc^ profiling by genotype revealed that the profiles did not differ between LF_141_ and FF_141_ sheep, which was characterised by the predominance of intraneuronal and intraglial PrP^sc^ and an absence of extracellular glia-associated PrP^sc^, which was in contrast to LL_141_ sheep (either VV or AA) where glia-associated PrP^sc^ was more prominent (see Fig. 3). These findings were consistent with results from studies in naturally infected sheep or sheep with scrapie induced by experimental cross-genotype transmission, which suggested that PrP^sc^ profiles were influenced by *PRNP* genotypes although other factors, such as source of infection and survival time could also be contributory [6, 35].

There is evidence from studies in experimentally and naturally infected sheep with scrapie that there is poor association between the severity of clinical signs and the level of PrP^res^ [36] or PrP^sc^ in the brain [37–39]. This was confirmed in the present study, using an established PrP^sc^ profiling method and detailed and regular clinical examinations for signs of scrapie. If the large variation in the total magnitude of PrP^sc^ immunolabelling in some VV and LL_141_ sheep was due to the difference in survival times and the severity of clinical signs despite having been infected with identical volumes of milk or doses of brain from the same donor, sheep with lower total PrP^sc^ scores should have shorter survival times with less severe clinical signs. However, this was not consistently observed. For example, the survival time was not necessarily longer in sheep with a higher PrP^sc^ score (1452 was culled later than 1424 even though the total PrP^sc^ was two times higher in 1424). Studies on the clinical progression in naturally affected sheep with scrapie suggested that head tremor, ataxia, teeth grinding, loss of body condition and loss of a menace response occurred more frequently with disease progression [29, 40]. There was some indication that sheep with higher PrP^sc^ scores, which had been challenged with milk or brain from the same donor, presented with more severe clinical signs: both brain-challenged VV sheep with a lower PrP^sc^ score (1452 and 1425) were not ataxic and had a menace response unlike the other VV sheep (1424), which was ataxic, lost the menace response and had a considerably higher PrP^sc^ score. Similarly, the severity of ataxia was considered less in an LL_141_ sheep with low PrP^sc^ score (1294) compared to one with a high PrP^sc^ score (1293), which also lost its appetite. However, two other VV sheep had almost identical signs (both ataxic with head tremor) despite considerable total PrP^sc^ score differences and whilst one (1451) was also teeth grinding, the other (1419) displayed signs of disequilibrium, which suggested more severe gait abnormalities.

The differences in the PrP^sc^ profiles in LL_141_ sheep challenged with milk from different donors may be the results of possible strain differences although it does not explain why the same phenomenon was not observed in LF_141_ sheep unless the wild-type alleles (LL_141_) produce greater phenotypic variability.

Similarly, the differences in the PrP^sc^ profiles in VV and LL_141_ sheep challenged with brain and milk from the same goat respectively may be to some extent explained by the different survival time and clinical severity of the sheep, which may alter the proportional localisation of PrP^sc^ immunolabelling so that sheep with longer survival times and more severe clinical signs have proportionally more intraneuronal and intraglial and less glia-associated immunolabelling, possibly as a result of progressive removal and internalization of glia-associated PrP^sc^ as the disease progresses (see Fig. 4: LL_141_ sheep 1293 had a longer survival time and more severe clinical signs than 1294). However, this does not quite explain the profile differences seen in VV sheep where the profiles were similar in sheep with the shortest and longest survival time (see Fig. 4: VV sheep 1418 and 1425 had the shortest survival time and a similar profile to 1451 and 1452 respectively, which had the longest survival time). It also does not explain why LF_141_ and FF_141_ sheep with comparatively longer survival times than LL_141_ sheep accumulate most PrP^sc^ intracellularly and lack glia-associated PrP^sc^, which suggests that PrP^sc^ is more prone to be internalized in neurons and not deposited in the membrane of glial cells in sheep carrying the F_141_ allele.

Discriminatory IHC using six different antibodies with different affinity to PrP^sc^ sequences is based on the observation that truncation of the prion protein of different strains (classical scrapie, classical ovine BSE and CH1641) is dependent on tissue, cell type and location within the tissue (intra- or extracellular). Extracellular PrP^sc^, which is not truncated, should be detected similarly with all antibodies whereas intracellular PrP^sc^ is truncated and therefore some antibodies (raised against the globular and C domains) should detect it while others (raised against the N terminus) may not. Whilst discriminatory IHC applied to lymphoid tissue agreed with the molecular profile obtained from lymphoid tissue, which identified the samples as consistent with classical scrapie, tests applied to CNS tissue produced a more varied result, which was dependent on *PRNP* polymorphisms at codon 141. This particularly affected the detection by N-terminal antibodies, such as 12B2 or P4. The magnitude of PrP^sc^ immunolabelling with the 12B2 antibody was reduced in sheep carrying the F_141_ allele compared to LL_141_ sheep, which was particularly striking for extracellular and intraglial PrP^sc^. Similarly, P4 antibody reactivity in the WB was reduced in brains from LF_141_ and particularly FF_141_ sheep. This antibody is used for the discriminatory WB to distinguish classical scrapie from BSE because brains from classical bovine BSE and experimental classical ovine BSE cases show no and greatly reduced signal, respectively [41]. LL_141_ sheep, including VV sheep, were classified as classical scrapie because PrP^res^ was detected with antibody P4, and the Sha31/P4 antibody ratio was as expected for classical scrapie isolates, whereas LF_141_ sheep produced an intermediate BSE-like profile with reduced detection with P4, and FF_141_ sheep produced a BSE-like profile with little or no PrP^res^ detection with P4 and the Sha31/P4 antibody ratio was unlike classical scrapie for both genotypes.

The results obtained from the discriminatory tests suggested that proteinase K used for WB cleaves brain samples from sheep with the L_141_ allotype towards the N terminus from the P4 epitope, thus resulting in PrP^res^ detection by both Sha31 and P4. In contrast, proteinase K appeared to cleave the F_141_ PrP protein towards the C terminus from the P4 epitope, possibly around the 141 position, thus resulting in detection by Sha31 (aa residue binding sites 148–155, see Figure 5) but not by P4 (aa 93–99). As a consequence, in LF_141_ sheep the P4 antibody only detects the L_141_ but not the F_141_ allotype resulting in decreased signal and in FF_141_ sheep all prion protein is of the F_141_ allotype and therefore there is almost no PrP^res^ signal with P4. The discriminatory IHC detected intraneuronal and intraglial PrP^sc^ with antibodies 12B2 and 521.7 in LF_141_ and FF_141_ sheep, which was unlike BSE, although the amount detected is lower by comparison with R145 and such a decrease was not seen in LL_141_ sheep. This suggests some cleavage of F_141_ allotype PrP^sc^ by cellular enzymes but such cleavage is not as drastic as with proteinase K seen in the WB. The decrease in intracellular PrP^sc^ immunolabelling with N terminal antibodies, which was higher in LF_141_ than FF_141_ sheep, remains unexplained unless some sort of interaction between the two allotypes makes the protein more susceptible to digestion.

The WB results highlight the difficulty in interpreting the discriminatory WB by examination of brain in cross-species transmission experiments because LF_141_ and particularly FF_141_ sheep demonstrated a classical BSE-like profile, even though there was sufficient evidence that the isolate was not BSE: i) LL_141_ sheep challenged with the same isolate produced a profile consistent with classical scrapie; ii) testing of lymphoid tissue produced a molecular and pathological phenotype consistent with classical scrapie; iii) the discriminatory IHC was unlike BSE and iv) the lack of pruritus in LF_141_ and FF_141_ was inconsistent with the clinical phenotype reported for classical BSE in sheep [19]. There is currently no evidence that this poses a problem for potential misdiagnosis during discriminatory testing of positive small ruminant field cases in accordance with current European Union legislation if similar cross-species transmission occurred naturally but the process of referral for a case giving an initial BSE-like characteristic initiates further investigative testing that would rule out BSE.

## Conclusions

In summary, this study provided evidence that the clinical, pathological and molecular phenotype of classical scrapie transmitted from goats to sheep is affected by *PRNP* polymorphisms, particularly at codon 141, which has to be taken into account when investigating strain characteristics by laboratory methods.

## Additional files

Additional file 1: study design. This file provides a graphical overview of the design of the pilot and milk transmission studies. (PDF 197 kb)

Additional file 4: clinical and pathological data. This file provides more detail about the clinical presentation, including scratch test score, and shows the individual PrP^sc^ scores to create the brain PrP^sc^ profiles as well as the scores for central nervous and lymphoid tissues obtained on the discriminatory IHC. (XLSX 38 kb)

## Abbreviations

AA: AA_136_RR_154_QQ_171_
aa: amino acid
BSE: bovine spongiform encephalopathy
CNS: central nervous system
F: female
FF_141_: AA_136_FF_141_RR_154_QQ_171_
IHC: immunohistochemistry
LF_141_: AA_136_LF_141_RR_154_QQ_171_
LL_141_: AA_136_LL_141_RR_154_QQ_171_
M/N: male neutered
*PRNP*: prion protein gene
PrP^res^: proteinase-resistant form of PrP^sc^
PrP^sc^: scrapie-associated prion protein
RAMALT: recto-anal mucosa-associated lymphoid tissue
SD: standard deviation
TSE: transmissible spongiform encephalopathy
UK: United Kingdom
VV: VV_136_RR_154_QQ_171_
WB: Western immunoblot
