# Supplementary figures and images for: Codon 141 polymorphisms of the ovine prion protein gene affect the phenotype of classical scrapie transmitted from goats to sheep
Source: BMC Vet Res. 2017 May 4;13:122. doi: 10.1186/s12917-017-1036-1 (PMC5418773; doi:10.1186/s12917-017-1036-1)

# Study design

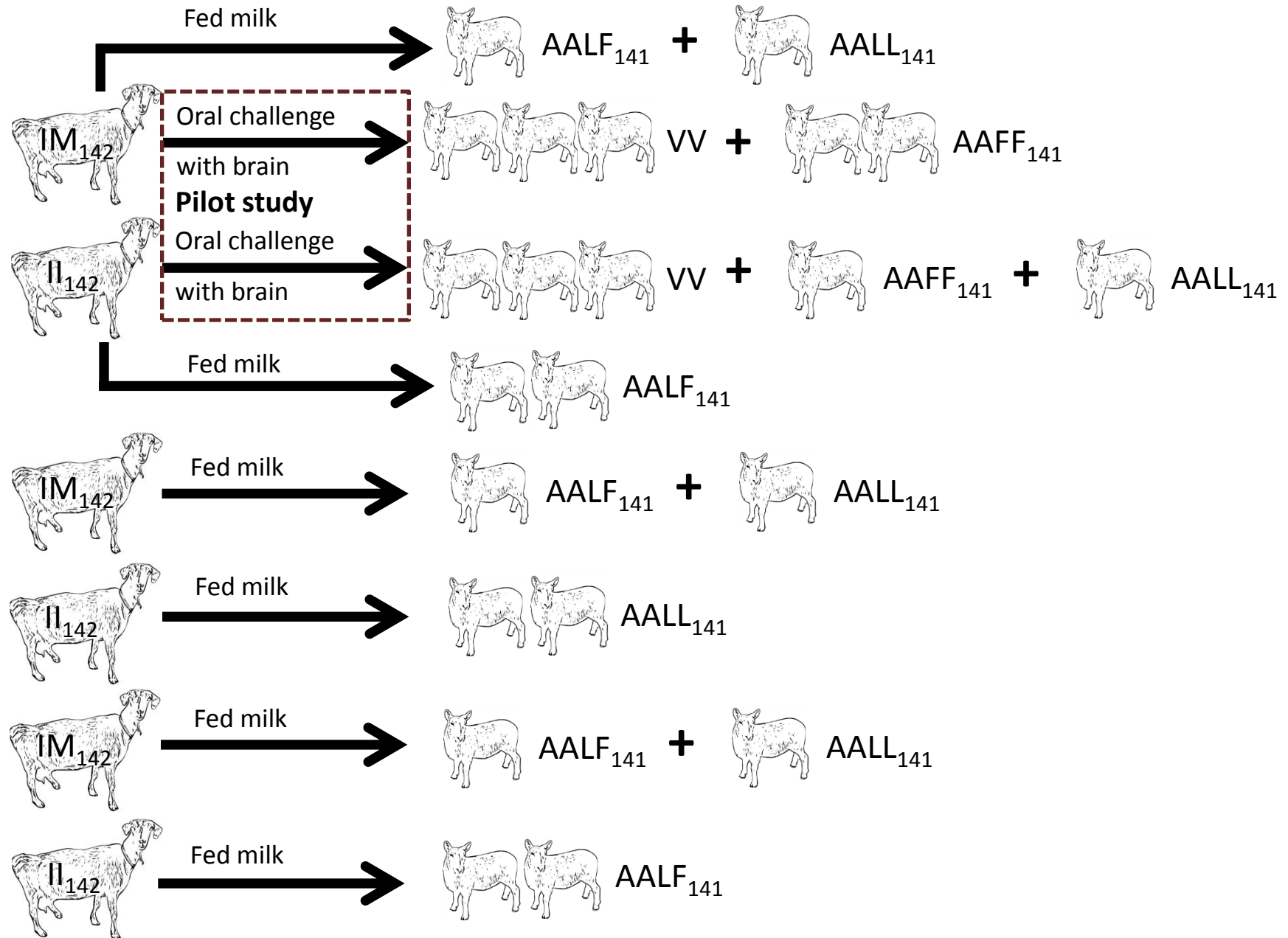

Supplement: Supplementary file 3 — study design. This file provides a graphical overview of the design of the pilot and milk transmission studies. (PDF 197 kb) [file 12917_2017_1036_MOESM1_ESM.pdf]
